# Development and validation of a risk prediction model for myocardial hypoperfusion after primary PCI in ST-segment elevation myocardial infarction

**DOI:** 10.3389/fcvm.2026.1806053

**Published:** 2026-04-21

**Authors:** Yan Zhao, Xiaoxia Fang, Huilin Li, Minglei Han, Fucheng Zhang, Mingming Qiao

**Affiliations:** 1 East Medical Imaging Department DSA (Catheter) Operating Room, Xinxiang Central Hospital, The Fourth Clinical College of Henan Medical University, Xinxiang, China; 2 Department of Nursing, Xinxiang Central Hospital, The Fourth Clinical College of Henan Medical University, Xinxiang, China; 3 Xinxiang Key Laboratory for Elderly Health Care and Promotion, The Fourth Clinical College of Henan Medical University, Xinxiang, China; 4 East Campus Department of Cardiovascular Medicine, Xinxiang Central Hospital, The Fourth Clinical College of Henan Medical University, Xinxiang, China

**Keywords:** myocardial hypoperfusion, nomogram, percutaneous coronary intervention, risk prediction model, ST-segment elevation myocardial infarction

## Abstract

**Objective:**

To analyze the determinants of myocardial hypoperfusion following primary percutaneous coronary intervention (PCI) in patients with acute ST-segment elevation myocardial infarction (STEMI) and to develop a risk prediction model.

**Methods:**

Clinical data from 434 patients with STEMI who underwent primary PCI at our hospital between January 2023 and June 2025 were retrospectively collected. Patients were randomly assigned to a training cohort (*n* = 304) and a validation cohort (*n* = 130) at a 7:3 ratio. Based on postprocedural myocardial perfusion, the training cohort was further divided into a hypoperfusion group (*n* = 103) and a normal perfusion group (*n* = 201). Candidate variables were screened using Boruta and least absolute shrinkage and selection operator (LASSO) regression, followed by multivariable logistic regression to identify independent predictors. A risk prediction model was constructed using R software and visualized as a nomogram. Model performance and clinical utility were evaluated using receiver operating characteristic (ROC) curves, calibration curves, and decision curve analysis (DCA).

**Results:**

Multivariable logistic regression identified time from onset to primary PCI, atorvastatin dose before PCI, balloon deflation method during PCI, red cell distribution width (RDW), and monoamine oxidase (MAO) levels as independent predictors of myocardial hypoperfusion (all *P* < 0.05). The nomogram demonstrated good discrimination, with area under the curve (AUC) values of 0.855 (95% CI: 0.811–0.900) in the training cohort and 0.838 (95% CI: 0.764–0.912) in the validation cohort. Calibration curves indicated good agreement between predicted and observed outcomes. Decision curve analysis showed that the model provided greater net benefit than both treat-all and treat-none strategies across threshold probabilities of 0.01–0.99 in the training cohort and 0.07–0.99 in the validation cohort.

**Conclusion:**

Time from onset to primary PCI, atorvastatin dose before PCI, balloon deflation method during PCI, RDW, and MAO levels are important determinants of myocardial hypoperfusion following primary PCI in patients with STEMI. The proposed prediction model demonstrated favorable predictive performance and clinical utility, suggesting its potential value for early risk stratification.

## 1 Introduction

Acute ST-segment elevation myocardial infarction (STEMI) is one of the most severe clinical manifestations of coronary artery disease and remains a leading cause of morbidity and mortality worldwide, posing a substantial burden on healthcare systems (1). Rapid restoration of coronary blood flow is critical for limiting infarct size and improving survival. Currently, primary percutaneous coronary intervention (PCI) is the preferred reperfusion strategy for patients with STEMI, as it enables prompt and sustained reopening of the infarct-related artery, enhances myocardial salvage, and significantly reduces mortality when performed in a timely manner (2, 3). However, successful recanalization of the epicardial coronary artery does not always translate into adequate myocardial tissue perfusion. A notable proportion of patients experience impaired microvascular blood flow following PCI, resulting in myocardial hypoperfusion. This phenomenon is increasingly recognized as an important determinant of prognosis, as it can hinder myocardial recovery, delay improvement in left ventricular function, and contribute to adverse cardiovascular outcomes such as heart failure, malignant arrhythmias, recurrent myocardial infarction, and elevated long-term mortality (4). The underlying mechanisms are complex and may involve distal embolization, ischemia–reperfusion injury, endothelial dysfunction, inflammatory responses, and microvascular obstruction (5).

Given its strong association with poor clinical outcomes, early identification of patients at high risk for myocardial hypoperfusion is essential for optimizing therapeutic strategies and improving prognosis. Importantly, preventive interventions implemented before or during PCI may provide greater clinical benefit than treatments initiated after microvascular damage has already occurred. Nevertheless, the determinants of myocardial hypoperfusion after primary PCI in STEMI have not been fully elucidated. Most previous studies have focused on single biomarkers or isolated clinical variables, which limits their ability to support comprehensive risk assessment and individualized clinical decision-making (6, 7). A robust and practical predictive tool integrating multiple risk factors could therefore facilitate early risk stratification and guide targeted preventive strategies in routine clinical practice. Accordingly, this study sought to identify the determinants of myocardial hypoperfusion after primary PCI in patients with STEMI and to develop a clinically applicable risk prediction model to enable early risk assessment and support precision clinical management.

## 2 Objects and methods

### 2.1 Sample size determination and study population

The sample size was calculated using the formula $N=Z_{\alpha }^{2}\times P(1-P)/\delta^{2}$, where P represents the risk of myocardial hypoperfusion after primary PCI in patients with STEMI. Prior to the formal study, we conducted a preliminary retrospective analysis of STEMI patients who underwent primary PCI at our center between January 2022 and December 2022. Among these patients, myocardial hypoperfusion occurred in 35 of 115 cases, corresponding to an incidence of 30.44%. This estimated event rate was used as the expected proportion (P) in the sample size calculation. The allowable error (*δ*) was set at 0.05. A two-sided test was used with a significance level of *α* = 0.05, corresponding to a *Z_α_* value of 1.960 according to the standard normal distribution table. The minimum required sample size was therefore calculated to be 325 patients. Considering an anticipated dropout rate of approximately 10%, and to ensure an adequate sample size for subsequent multivariable logistic regression analysis, a total of 434 patients were ultimately included in the study.

**Figure 1:**
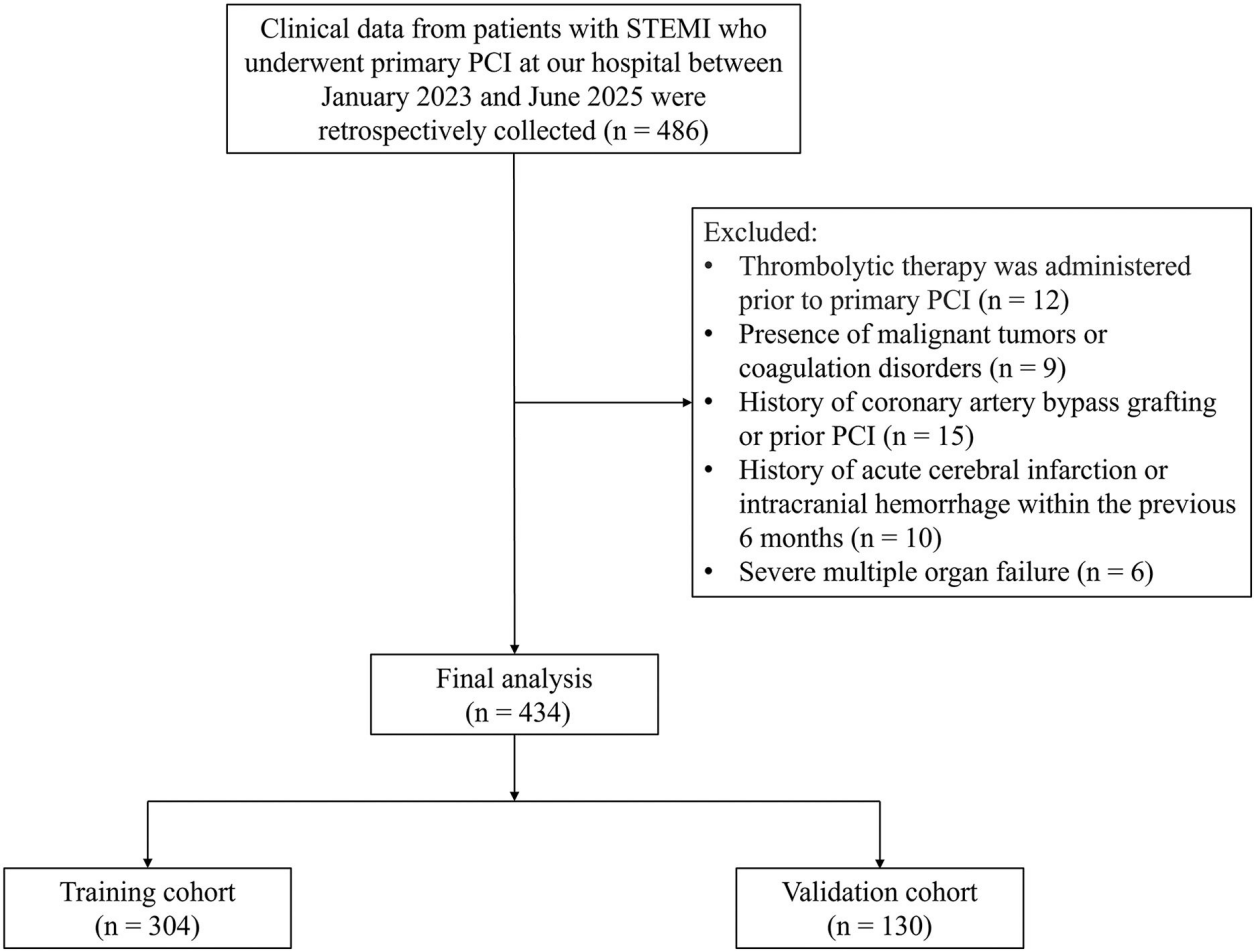
Flowchart of patient selection in this retrospective study, illustrating the screening process and final inclusion of 434 patients with STEMI. PCI, percutaneous coronary intervention; STEMI, ST-segment elevation myocardial infarction.

Clinical data from 434 patients with STEMI who underwent primary PCI at our hospital between January 2023 and June 2025 were retrospectively collected. The patients were randomly assigned in a 7:3 ratio to a training cohort (*n* = 304) and a validation cohort (*n* = 130). Myocardial hypoperfusion after primary PCI occurred in 103 patients in the training cohort and 44 patients in the validation cohort. Inclusion criteria: (i) Patients met the diagnostic criteria for STEMI (8) and were experiencing their first onset; (ii) Primary PCI was performed by the same team of experienced physicians; (iii) Time from symptom onset to primary PCI was <12 h; and (iv) Complete clinical data were available. Exclusion criteria were: (i) Thrombolytic therapy was administered prior to primary PCI; (ii) Presence of malignant tumors or coagulation disorders; (iii) History of coronary artery bypass grafting or prior PCI; (iv) History of acute cerebral infarction or intracranial hemorrhage within the previous 6 months; (v) Severe multiple organ failure. A flowchart detailing the patient selection process is presented in Figure 1.

### 2.2 Data collection

#### 2.2.1 Baseline characteristics

Clinical data were retrieved from the hospital electronic medical record system, and all relevant variables were systematically recorded and organized for analysis. Baseline characteristics included age, sex (male or female), comorbidities (hypertension, diabetes mellitus, and angina pectoris), and lifestyle factors (drinking and smoking status). In addition, the infarct-related artery (left main coronary artery, left anterior descending artery, left circumflex artery, or right coronary artery), time from onset to primary PCI, and preprocedural cardiac function assessed using the Killip classification (9) (Class I–II vs. Class III–IV) were collected.

#### 2.2.2 Periprocedural characteristics

Periprocedural variables included procedure duration, maximum stent diameter, atorvastatin dose before PCI (80 mg or 40 mg), and the balloon deflation method during PCI (slow deflation vs. conventional deflation).

#### 2.2.3 Laboratory parameters before primary PCI

Laboratory parameters obtained prior to primary PCI included routine blood indices (white blood cell count [WBC], hemoglobin [Hb], platelet count [PLT], and red cell distribution width [RDW]); lipid profile (total cholesterol [TC], low-density lipoprotein cholesterol [LDL-C], high-density lipoprotein cholesterol [HDL-C], and triglycerides [TG]); biomarkers of myocardial injury [serum creatinine [Scr], cardiac troponin I [cTnI], and creatine kinase-MB [CK-MB]]; and monoamine oxidase (MAO) levels.

### 2.3 Definition of myocardial hypoperfusion after primary PCI

Myocardial perfusion was assessed in all patients after primary PCI using coronary angiography. Blood flow was graded according to the Thrombolysis in Myocardial Infarction (TIMI) flow classification (10): Grade 0 (no perfusion), no antegrade flow beyond the site of occlusion; Grade I (penetration without perfusion), contrast passes beyond the obstruction but fails to opacify the distal coronary bed; Grade II (partial perfusion), contrast opacifies the distal vessel but at a delayed rate compared with normal coronary arteries; and Grade III (complete perfusion), normal flow with rapid filling and clearance of contrast. Patients with a postprocedural TIMI flow grade <III were classified into the hypoperfusion group, whereas those with TIMI grade III were assigned to the normal perfusion group.

### 2.4 Statistical analysis

Categorical variables are presented as frequencies (percentages) and were compared using the chi-square test. Continuous variables with a normal distribution are expressed as the mean ± standard deviation (SD) and were analyzed using the independent-samples *t*-test. Feature selection was conducted using both the least absolute shrinkage and selection operator (LASSO) regression and the Boruta algorithm. Continuous variables were retained as continuous measurements and were standardized using z-score normalization (mean-centered and scaled by the standard deviation) prior to LASSO regression to account for differences in measurement scales. Only variables identified by the intersection of these two methods were retained to enhance model stability and reduce the risk of overfitting. Multicollinearity among candidate predictors was evaluated using the variance inflation factor (VIF) and tolerance, with VIF >5 and/or tolerance <0.10 indicating significant collinearity. Variables without significant collinearity were subsequently entered into the multivariable logistic regression model to identify independent predictors.

A risk prediction model was developed based on the independent predictors, and a nomogram was constructed to provide a visualized risk estimation tool. Model discrimination was assessed using the receiver operating characteristic (ROC) curve, while calibration was evaluated with a calibration curve. Decision curve analysis (DCA) was performed to determine the clinical net benefit of the model. All statistical analyses were performed using SPSS version 23.0 and R software version 4.3.3. A two-sided *P* value < 0.05 was considered statistically significant.

## 3 Results

### 3.1 Baseline data of the training and validation cohorts

In this study, 304 patients were assigned to the training cohort and 130 to the validation cohort. The mean age was 58.06 ± 5.93 years in the training cohort and 58.12 ± 5.79 years in the validation cohort, with males comprising 67.43% and 66.02% of the cohorts, respectively. No significant differences were observed between the two cohorts in baseline characteristics, procedural variables, or laboratory parameters (all *P* > 0.05), indicating good comparability between the groups (Table 1).

**Table 1:** Comparison of clinical data between the training and validation cohorts.

Variables	Training cohort (*n* = 304)	Validation cohort (*n* = 130)	*χ*2/*t*	*P*
Age (years)	58.06 ± 5.93	58.12 ± 5.79	0.097	0.923
Sex, *n* (%)				
Male	205 (67.43)	87 (66.92)	0.011	0.917
Female	99 (32.57)	43 (33.08)		
Comorbid chronic diseases, *n* (%)				
Hypertension	84 (27.63)	39 (30.00)	0.251	0.616
Diabetes mellitus	64 (21.05)	27 (20.77)	0.004	0.949
Angina pectoris	108 (35.53)	46 (35.38)	0.001	0.974
Unhealthy lifestyle habits, *n* (%)				
Drinking	113 (37.17)	44 (33.85)	0.436	0.509
Smoking	139 (45.72)	59 (45.38)	0.004	0.950
Infarct-related artery, *n* (%)				
Left main coronary artery	4 (1.32)	3 (2.31)	1.083	0.781
Left anterior descending artery	164 (53.95)	73 (56.15)		
Left circumflex artery	50 (16.45)	18 (13.85)		
Right coronary artery	86 (28.29)	36 (27.69)		
Time from onset to primary PCI (h)	6.53 ± 1.78	6.48 ± 1.77	0.269	0.788
Killip class before PCI, *n* (%)				
Class I–II	219 (72.04)	90 (69.23)	0.350	0.551
Class III–IV	85 (27.96)	40 (30.77)		
Procedure time (min)	79.55 ± 11.13	79.02 ± 11.14	0.454	0.650
Maximum stent diameter (mm)	4.01 ± 1.08	3.88 ± 0.97	1.183	0.237
Atorvastatin dose before PCI, *n* (%)				
80 mg	155 (50.99)	70 (53.85)	0.298	0.585
40 mg	149 (49.01)	60 (46.15)		
Balloon deflation method during PCI, *n* (%)				
Slow deflation	136 (44.74)	53 (40.77)	0.583	0.445
Conventional deflation	168 (55.26)	77 (59.23)		
WBC (×10^9^·L−^1^)	9.63 ± 1.72	9.69 ± 1.76	0.331	0.741
Hb (g·L−^1^)	144.13 ± 13.55	143.23 ± 13.75	0.631	0.528
PLT (×10^9^·L−^1^)	201.18 ± 21.95	199.53 ± 23.10	0.706	0.481
RDW (%)	13.57 ± 1.37	13.66 ± 1.35	0.630	0.529
TC (mmol·L−^1^)	5.06 ± 0.79	5.01 ± 0.82	0.597	0.551
LDL-C (mmol·L−^1^)	2.52 ± 0.48	2.50 ± 0.48	0.398	0.691
HDL-C (mmol·L−^1^)	1.17 ± 0.34	1.18 ± 0.33	0.283	0.777
TG (mmol·L−^1^)	1.38 ± 0.52	1.37 ± 0.53	0.183	0.855
Scr (*μ*mol·L−^1^)	77.05 ± 10.09	75.96 ± 9.61	1.046	0.296
CTnI (μg·L^−1^)	0.36 ± 0.08	0.35 ± 0.07	1.237	0.217
CK-MB (μg·L^−1^)	13.11 ± 2.77	12.99 ± 2.97	0.405	0.686
MAO (U·L−^1^)	10.85 ± 2.27	10.89 ± 2.32	0.167	0.867

PCI, percutaneous coronary intervention; WBC, white blood cell count; Hb, hemoglobin; PLT, platelet count; RDW, red cell distribution width; TC, total cholesterol; LDL-C, low-density lipoprotein cholesterol; HDL-C, high-density lipoprotein cholesterol; TG, triglycerides; Scr, serum creatinine; cTnI, cardiac troponin I; CK-MB, creatine kinase-MB isoenzyme; MAO, monoamine oxidase.

### 3.2 Comparison of clinical data between the hypoperfusion and normal perfusion groups in the training cohort

Among the training cohort, 103 patients were classified into the hypoperfusion group and 201 into the normal perfusion group. Compared with the normal perfusion group, patients in the hypoperfusion group had a higher prevalence of angina pectoris, longer time from onset to primary PCI, higher Killip class, lower use of 80 mg atorvastatin before PCI, and less frequent slow balloon deflation (all *P* < 0.05). In addition, RDW, cTnI, and MAO levels were significantly higher in the hypoperfusion group than in the normal perfusion group (all *P* < 0.05). No significant differences were observed in other variables (all *P* > 0.05) (Table 2).

**Table 2:** Comparison of clinical characteristics between the hypoperfusion group and the normal perfusion group.

Variables	Hypoperfusion group (*n* = 103)	Normal perfusion group (*n* = 201)	*χ*^2^/*t*	*P*
Age (years)	58.61 ± 5.33	57.78 ± 6.21	1.157	0.248
Sex, *n* (%)				
Male	73 (70.87)	132 (65.67)	0.839	0.360
Female	30 (29.13)	69 (34.33)		
Comorbid chronic diseases, *n* (%)				
Hypertension	29 (28.16)	55 (27.36)	0.021	0.884
Diabetes mellitus	24 (23.30)	40 (19.90)	0.474	0.491
Angina pectoris	45 (43.69)	63 (31.34)	4.532	0.033
Unhealthy lifestyle habits, *n* (%)				
Drinking	42 (40.78)	71 (35.32)	0.867	0.352
Smoking	50 (48.54)	89 (44.28)	0.499	0.480
Infarct-related artery, *n* (%)				
Left main coronary artery	1 (0.97)	3 (1.49)	0.562	0.937
Left anterior descending artery	57 (55.34)	107 (53.23)		
Left circumflex artery	18 (17.48)	32 (15.92)		
Right coronary artery	27 (26.21)	59 (29.35)		
Time from onset to primary PCI (h)	7.03 ± 1.87	6.27 ± 1.68	3.587	<0.001
Killip class before PCI, *n* (%)				
Class I–II	64 (62.14)	155 (77.11)	7.585	0.006
Class III–IV	39 (37.86)	46 (22.89)		
Procedure time (min)	80.85 ± 12.15	78.88 ± 10.54	1.400	0.163
Maximum stent diameter (mm)	3.93 ± 1.02	4.05 ± 1.10	0.918	0.359
Atorvastatin dose before PCI, *n* (%)				
80 mg	36 (34.95)	119 (59.20)	16.029	<0.001
40 mg	67 (65.05)	82 (40.80)		
Balloon deflation method during PCI, *n* (%)				
Slow deflation	34 (33.01)	102 (50.75)	8.666	0.003
Conventional deflation	69 (66.99)	99 (49.25)		
WBC (×10^9^·L−^1^)	9.81 ± 1.77	9.53 ± 1.69	1.344	0.180
Hb (g·L−^11^)	142.01 ± 15.51	145.21 ± 12.33	1.820	0.071
PLT (×10^9^·L−^1^)	198.76 ± 23.08	202.41 ± 21.29	1.374	0.170
RDW (%)	14.65 ± 1.27	13.02 ± 1.05	11.188	<0.001
TC (mmol·L−^1^)	5.12 ± 0.85	5.03 ± 0.76	0.940	0.348
LDL-C (mmol·L−^1^)	2.58 ± 0.42	2.49 ± 0.51	1.619	0.107
HDL-C (mmol·L−^1^)	1.12 ± 0.30	1.20 ± 0.36	1.926	0.055
TG (mmol·L−^1^)	1.42 ± 0.55	1.35 ± 0.51	1.171	0.243
Scr (μmol·L−^1^)	78.21 ± 10.33	76.45 ± 9.94	1.442	0.150
CTnI (μg·L^−1^)	0.38 ± 0.10	0.35 ± 0.07	2.582	0.011
CK-MB (μg·L^−1^)	13.48 ± 3.15	12.91 ± 2.54	1.700	0.090
MAO (U·L−^1^)	12.21 ± 2.13	10.15 ± 2.01	8.278	<0.001

PCI, percutaneous coronary intervention; WBC, white blood cell count; Hb, hemoglobin; PLT, platelet count; RDW, red cell distribution width; TC, total cholesterol; LDL-C, low-density lipoprotein cholesterol; HDL-C, high-density lipoprotein cholesterol; TG, triglycerides; Scr, serum creatinine; cTnI, cardiac troponin I; CK-MB, creatine kinase-MB isoenzyme; MAO, monoamine oxidase.

### 3.3 Variable selection

**Figure 2:**
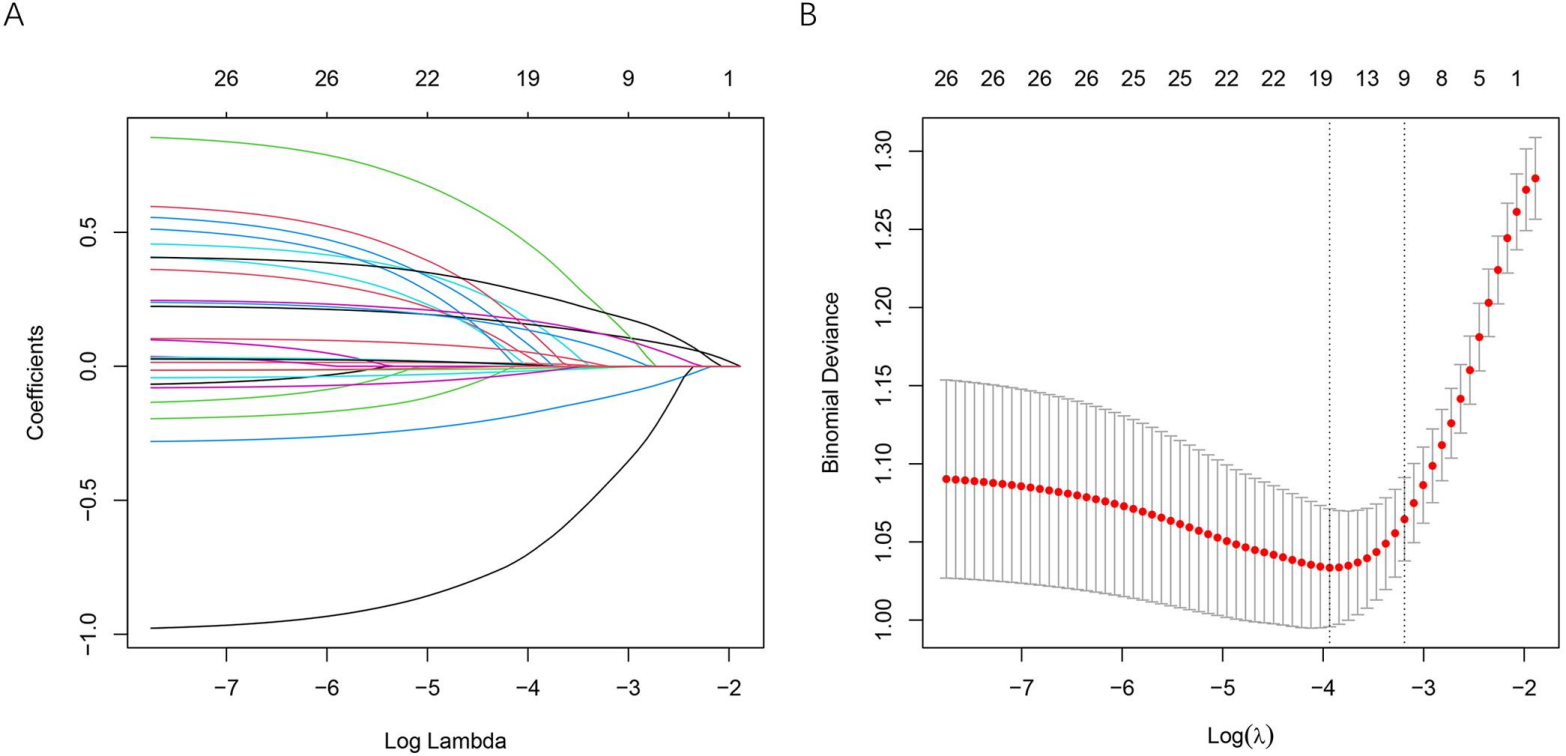
Variable selection using LASSO regression. **(A)** Coefficient profiles of candidate variables plotted against log(*λ*). Each curve represents a regression coefficient for an individual variable, and coefficients gradually shrink toward zero as the penalty increases, with some variables eliminated from the model. **(B)** Selection of the optimal penalty parameter (*λ*) using 10-fold cross-validation based on binomial deviance; the dotted vertical lines indicate the minimum criteria and the 1-SE criteria.

To identify candidate predictors of hypoperfusion, we applied LASSO regression and Boruta feature selection. In the LASSO model, coefficient trajectories are shown across different log(*λ*) values (Figure 2A). With increasing *λ*, most coefficients gradually shrank toward zero, indicating progressive elimination of less-informative variables. Ten-fold cross-validation was used to determine the optimal penalty (Figure 2B). At the *λ* corresponding to the minimum binomial deviance, a subset of variables with non-zero coefficients was retained (approximately 9 predictors), which were considered potential contributors to hypoperfusion.

**Figure 3:**
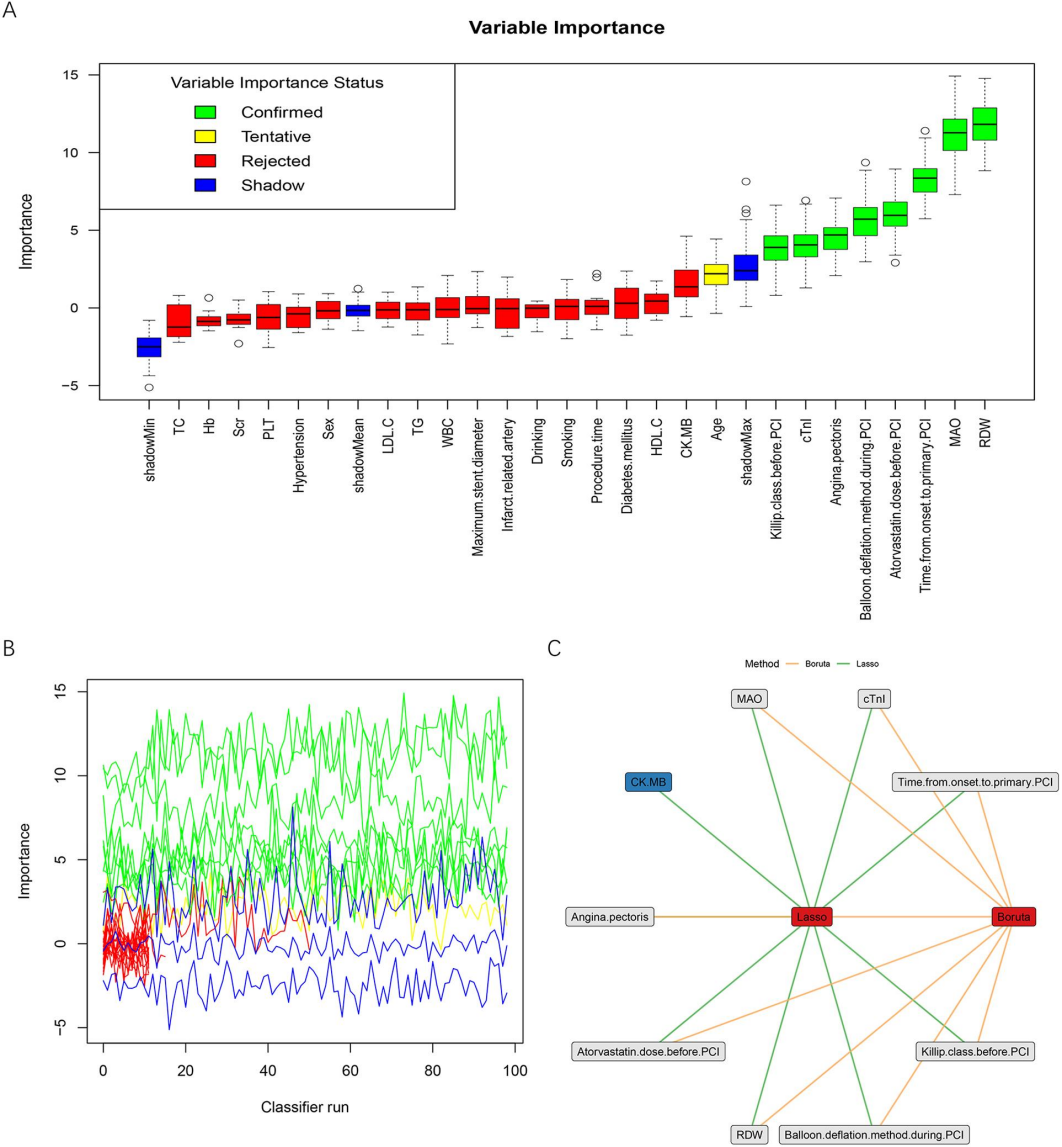
Variable importance and selection using boruta and LASSO. **(A)** Boxplots showing the distribution of importance scores for each variable. Green indicates confirmed important variables, yellow indicates tentative variables, red indicates rejected variables, and blue represents shadow features. **(B)** Importance of variables across Boruta iterations, demonstrating the stability of confirmed features compared with shadow attributes. **(C)** Overlap of variables selected by LASSO and Boruta, illustrating the final predictors included for model development. PCI, percutaneous coronary intervention; WBC, white blood cell count; Hb, hemoglobin; PLT, platelet count; RDW, red cell distribution width; TC, total cholesterol; LDL-C, low-density lipoprotein cholesterol; HDL-C, high-density lipoprotein cholesterol; TG, triglycerides; Scr, serum creatinine; cTnI, cardiac troponin I; CK-MB, creatine kinase-MB isoenzyme; MAO, monoamine oxidase.

Boruta feature selection was further applied to evaluate the importance of variables based on a random forest algorithm. The results identified several variables as important predictors, while others were rejected, thereby improving the reliability of feature selection (Figures 3A,B). The overlap between LASSO and Boruta included angina pectoris, time from onset to primary PCI, Killip class before PCI, atorvastatin dose before PCI, balloon deflation method during PCI, RDW, cTnI, and MAO (Figure 3C). These variables were ultimately selected for subsequent model development. The coding of these variables is presented in Table 3.

**Table 3:** Variable coding scheme.

Variables	Assignment description	Coding
Angina pectoris	Categorical variable	1 = Yes, 0 = No
Time from onset to primary PCI	Continuous variable	Measured value
Killip class before PCI	Categorical variable	1 = Class III–IV, 0 = Class I–II
Atorvastatin dose before PCI	Categorical variable	1 = 40 mg, 0 = 80 mg
Balloon deflation method during PCI	Categorical variable	1 = Conventional deflation, 0 = Slow deflation
RDW	Continuous variable	Measured value
cTnI	Continuous variable	Measured value
MAO	Continuous variable	Measured value

PCI, percutaneous coronary intervention; RDW, red cell distribution width; cTnI, cardiac troponin I; MAO, monoamine oxidase.

### 3.4 Multivariate logistic regression analysis of myocardial hypoperfusion

Variables identified by both LASSO and Boruta were included in the multivariate logistic regression analysis. Before modeling, collinearity diagnostics were performed, showing tolerance values between 0.887 and 0.979 and VIF values between 1.021 and 1.128, suggesting no significant multicollinearity among the variables (Table 4). The multivariate analysis revealed that longer time from onset to primary PCI (OR = 1.395, 95% CI: 1.135–1.716, *P* = 0.002), higher RDW (OR = 3.703, 95% CI: 2.598–5.278, *P* < 0.001), elevated MAO (OR = 1.665, 95% CI: 1.365–2.030, *P* < 0.001), use of 40 mg atorvastatin before PCI (OR = 2.328, 95% CI: 1.141–4.750, *P* = 0.020), and conventional balloon deflation during PCI (OR = 2.143, 95% CI: 1.018–4.509, *P* = 0.045) were independent risk factors for myocardial hypoperfusion. Angina pectoris, Killip class before PCI, and cTnI were not independently associated with myocardial hypoperfusion (*P* > 0.05). The detailed findings are shown in Table 4.

**Table 4:** Multivariate logistic regression analysis of myocardial hypoperfusion.

Variables	Collinearity diagnostics		Multivariable logistic regression					
	Tolerance	VIF	*β*	*SE*	*Waldχ* ^2^	*P*	*OR*	95%*CI*
Angina pectoris	0.947	1.056	0.516	0.383	1.813	0.178	1.675	0.791–3.547
Time from onset to primary PCI	0.979	1.021	0.333	0.105	9.968	0.002	1.395	1.135–1.716
Killip class before PCI	0.971	1.030	0.684	0.388	3.099	0.078	1.982	0.925–4.243
Atorvastatin dose before PCI	0.940	1.064	0.845	0.364	5.388	0.020	2.328	1.141–4.750
Balloon deflation method during PCI	0.940	1.064	0.762	0.380	4.031	0.045	2.143	1.018–4.509
RDW	0.915	1.093	1.309	0.181	52.426	<0.001	3.703	2.598–5.278
cTnI	0.977	1.023	0.401	0.215	3.488	0.062	1.494	0.980–2.275
MAO	0.887	1.128	0.510	0.101	25.396	<0.001	1.665	1.365–2.030

VIF, variance inflation factor; SE, standard error; OR, odds ratio; CI, confidence interval; PCI, percutaneous coronary intervention; RDW, red cell distribution width; cTnI, cardiac troponin I; MAO, monoamine oxidase.

### 3.5 Construction of a risk prediction model for myocardial hypoperfusion after primary PCI in patients with STEMI

**Figure 4:**
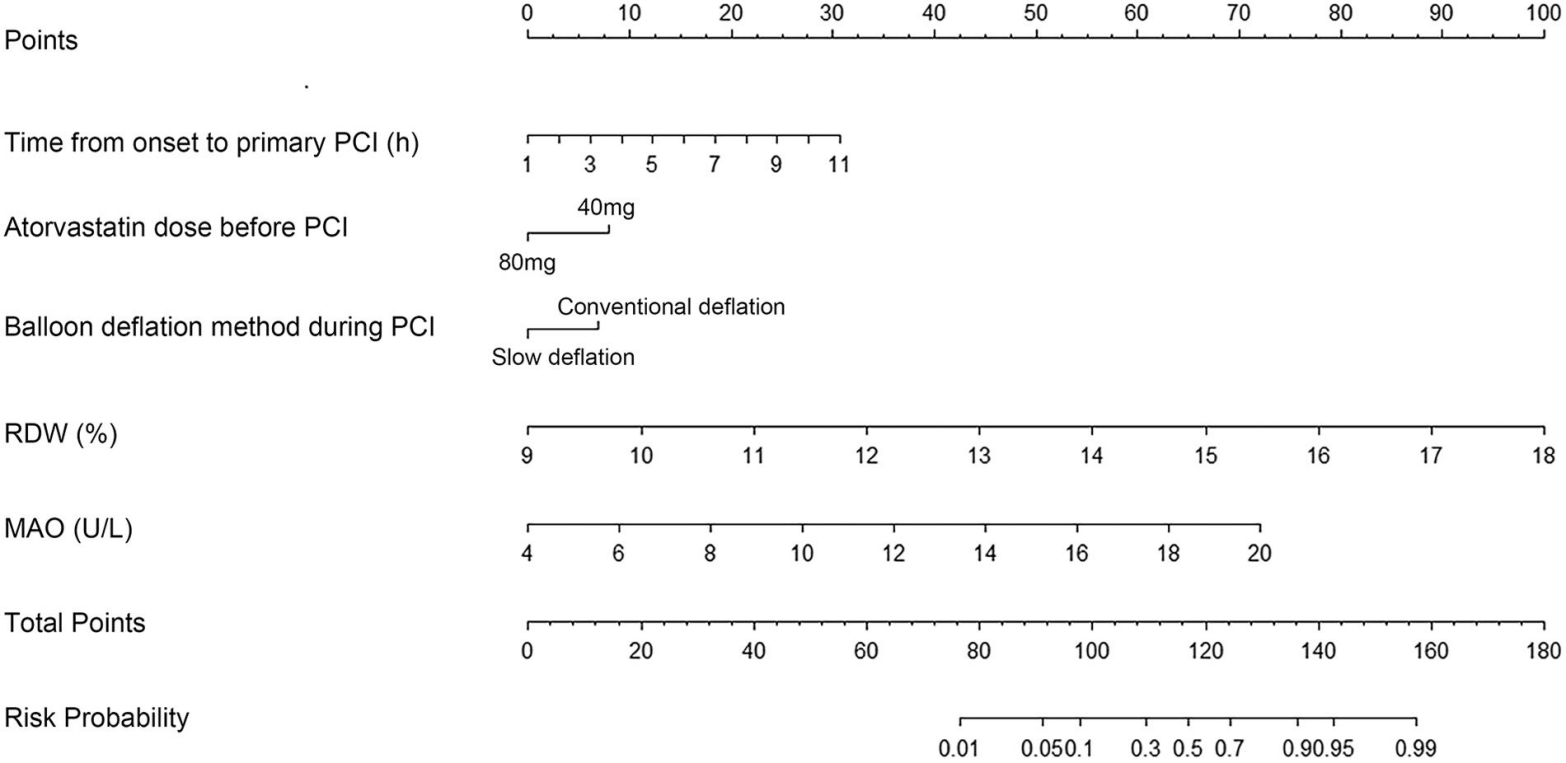
Nomogram for predicting the risk of myocardial hypoperfusion after primary PCI in patients with STEMI. PCI, percutaneous coronary intervention; RDW, red cell distribution width; MAO, monoamine oxidase.

Based on independent predictors identified through multivariable logistic regression, a risk prediction model for myocardial hypoperfusion following primary PCI in patients with STEMI was established and graphically represented as a nomogram using R software (Figure 4). To apply the nomogram, the value of each predictor is first projected onto the corresponding “Points” axis to obtain an individual score. The scores for all variables are then summed to determine the patient's total score on the “Total Points” axis. Finally, a vertical line is drawn downward from the total score to the “Predicted Probability” axis to estimate the individual risk of myocardial hypoperfusion following primary PCI.

### 3.6 Performance evaluation and validation of the risk prediction model

**Figure 5:**
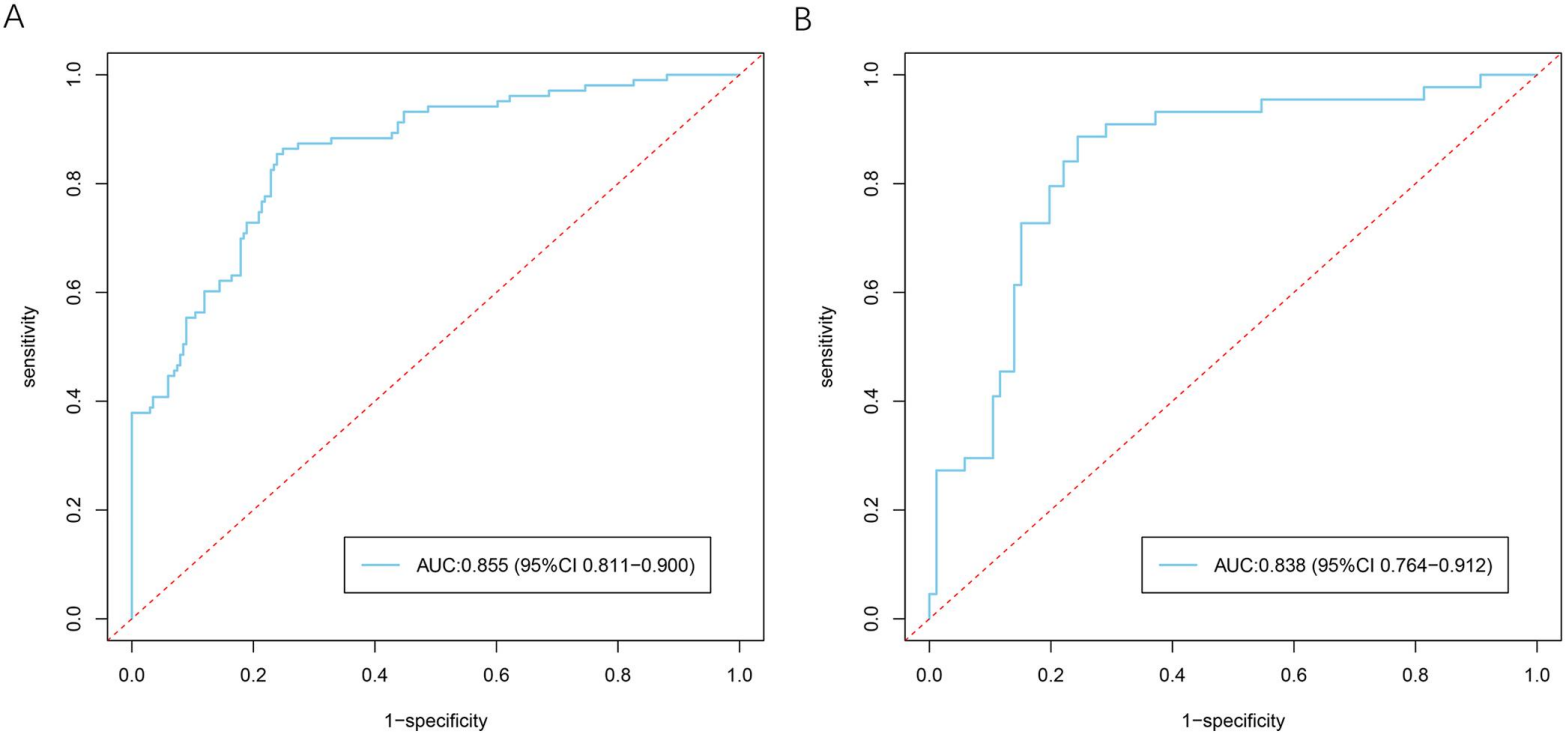
ROC curves of the risk prediction model for myocardial hypoperfusion after primary PCI in patients with STEMI. **(A)** ROC curve of the model in the training cohort. **(B)** ROC curve of the model in the validation cohort.

The predictive performance of the model was assessed in terms of discrimination and calibration in both the training and validation cohorts. ROC curve analysis demonstrated that the model had good discriminatory ability. In the training cohort, the area under the curve (AUC) was 0.855 (95% CI: 0.811–0.900), indicating strong predictive accuracy. Similarly, in the validation cohort, the model achieved an AUC of 0.838 (95% CI: 0.764–0.912), suggesting favorable performance and stability (Figures 5A,B).

**Figure 6:**
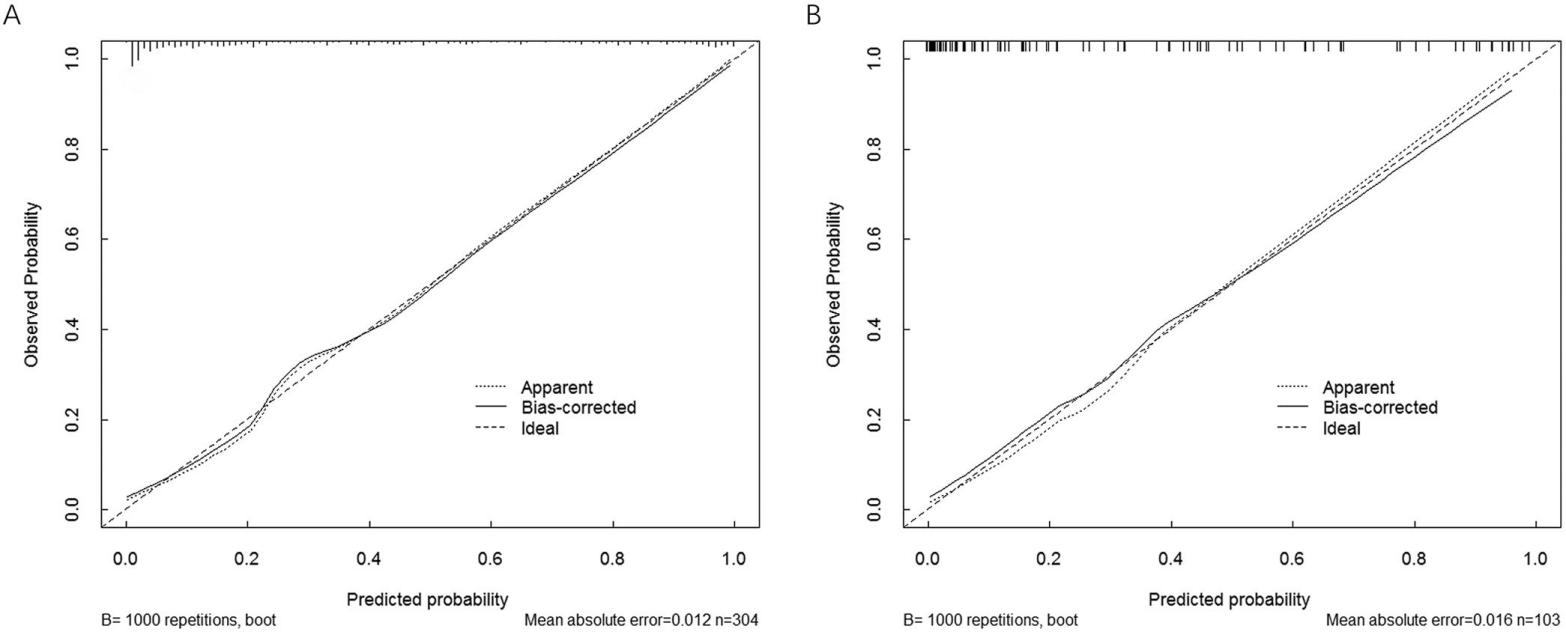
Calibration curves of the risk prediction model for myocardial hypoperfusion after primary PCI in patients with STEMI. **(A)** Calibration curve of the model in the training cohort. **(B)** Calibration curve of the model in the validation cohort.

Calibration curves were generated to evaluate the agreement between predicted and observed probabilities of myocardial hypoperfusion. The curves showed close alignment between the apparent and bias-corrected estimates and the ideal reference line in both cohorts. The Hosmer–Lemeshow goodness-of-fit test was performed to further evaluate model calibration by comparing the predicted probabilities with the observed outcomes. A non-significant result (*P* > 0.05) indicates good agreement between predicted and observed risks. In the present study, the Hosmer–Lemeshow test showed no significant difference in either the training cohort (*χ*^2^ = 6.862, *P* = 0.557) or the validation cohort (*χ*^2^ = 10.048, *P* = 0.262), indicating good calibration of the prediction model (Figures 6A,B). The mean absolute errors were 0.012 and 0.016 for the training and validation cohorts, respectively, further supporting the reliability of the model.

### 3.7 Clinical decision-making utility of the risk prediction model

**Figure 7:**
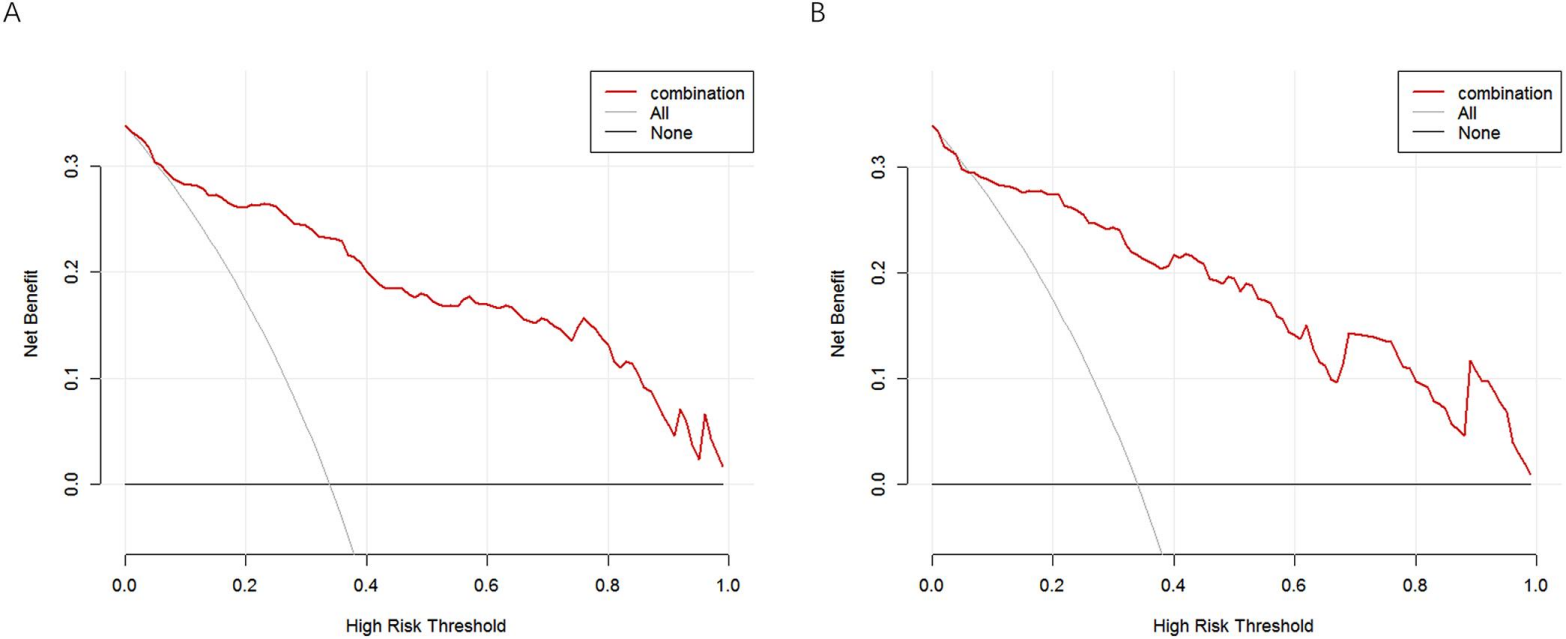
Decision curve analysis of the risk prediction model for myocardial hypoperfusion after primary PCI in patients with STEMI. **(A)** DCA of the prediction model in the training cohort. **(B)** DCA of the prediction model in the validation cohort.

DCA was performed to evaluate the clinical usefulness of the prediction model in both the training and validation cohorts. The results showed that the model provided a greater net benefit than the treat-all and treat-none strategies across a broad range of threshold probabilities. Specifically, in the training cohort, the model demonstrated superior net benefit when the threshold probability ranged from 0.01 to 0.99. Similarly, in the validation cohort, the prediction model remained clinically advantageous within a threshold probability range of 0.07–0.99 (Figures 7A,B). These findings indicate that the model may effectively support clinical decision-making by enabling improved risk stratification and facilitating targeted preventive interventions for patients at high risk of myocardial hypoperfusion following primary PCI.

## 4 Discussion

In the present study, we systematically identified the determinants of myocardial hypoperfusion following primary PCI in patients with STEMI and developed a nomogram-based prediction model to facilitate early risk stratification. Using the intersection of Boruta and LASSO feature selection, eight candidate variables were initially identified, and multivariable logistic regression ultimately retained five independent predictors—time from onset to primary PCI, atorvastatin dose before PCI, balloon deflation method during PCI, RDW, and MAO levels—which were incorporated into the final model. This stepwise approach enhanced model robustness while reducing the likelihood of overfitting. The model demonstrated favorable predictive performance, with AUC values of 0.855 and 0.838 in the training and validation cohorts, respectively, along with good calibration. Decision curve analysis further supported its clinical usefulness by showing a positive net benefit across a broad range of threshold probabilities. Notably, the predictors included in the model are routinely available in clinical practice, highlighting its practicality for real-world implementation. Overall, the proposed model provides a reliable tool for individualized risk assessment and may assist clinicians in identifying patients at elevated risk of myocardial hypoperfusion, thereby facilitating timely preventive strategies and potentially improving clinical outcomes.

Myocardial hypoperfusion is frequently observed following primary PCI in patients with STEMI, and affected individuals have a several-fold higher risk of mortality compared with those achieving normal myocardial perfusion, underscoring its pivotal role in adverse clinical outcomes (11, 12). Consequently, post-PCI myocardial hypoperfusion has emerged as a major focus of contemporary cardiovascular research. Elucidating its determinants is essential for optimizing clinical decision-making, improving myocardial reperfusion, and ultimately enhancing patient prognosis and quality of life (13). Previous nomogram studies on in-hospital adverse outcomes after PCI in patients with STEMI have mainly focused on major adverse cardiovascular events (MACE) or in-hospital cardiac arrest (IHCA), with relatively few directly targeting the phenotype of myocardial hypoperfusion. For example, Fang et al. developed a nomogram to predict in-hospital MACE after PCI in patients with STEMI, incorporating indicators such as Killip class, blood urea nitrogen, LVEF, and NT-proBNP (14). Another study established a nomogram for predicting IHCA after successful PPCI, including variables such as age, Killip class III–IV, systolic blood pressure, and moderate-to-severe calcification (15). Against this background, the present study identified five independent predictors of myocardial hypoperfusion following primary PCI. Understanding the potential mechanisms linking these factors to impaired myocardial perfusion may provide important insights into risk stratification and targeted prevention. Prolonged time from symptom onset to primary PCI may directly lead to ongoing myocardial necrosis. Necrotic cardiomyocytes release large amounts of inflammatory mediators, which activate neutrophils and promote their adhesion to the microvascular endothelium, resulting in capillary obstruction and structural damage. Consequently, even when the culprit artery is successfully reopened, adequate perfusion of the ischemic myocardium may not be fully restored, thereby increasing the risk of myocardial hypoperfusion (16). In addition, persistent myocardial ischemia can induce sustained platelet activation and promote the formation of microthrombi. These microthrombi are often not completely eliminated during PCI and may continue to obstruct the local vasculature, elevate microvascular resistance, and impair postprocedural myocardial blood flow. Scarsini et al. (17) further demonstrated that prolonged ischemia disrupts intracellular osmotic balance, leading to cellular edema. Severe myocardial edema may extend into the interstitial space, compress surrounding capillary beds, reduce microvascular blood volume, and ultimately increase microcirculatory resistance, thereby compromising myocardial reperfusion after PCI. Consistent with these findings, our results identified time from onset to primary PCI as an important determinant of myocardial hypoperfusion in patients with STEMI. Coronary microvascular dysfunction resulting from ischemic injury, distal embolization, and reperfusion injury is widely recognized as a central mechanism underlying myocardial hypoperfusion following primary PCI in patients with STEMI (5). Atorvastatin, a hydroxymethylglutaryl–coenzyme A reductase inhibitor widely used as first-line therapy, has been shown to reduce residual platelet activity by inhibiting platelet aggregation, thereby limiting thrombus formation and improving myocardial perfusion (18). Beyond its antithrombotic effects, atorvastatin may also alleviate myocardial metabolic disturbances and attenuate ischemia–reperfusion injury through modulation of inflammatory signaling pathways. Compared with the conventional 40-mg regimen, a higher loading dose of 80 mg may provide a more rapid and potent pharmacological effect, potentially enhancing reperfusion and reducing the risk of myocardial hypoperfusion (19). A randomized double-blind trial by Liakopoulos et al. (20) showed that high-dose atorvastatin administered before PCI reduced reperfusion injury and improved outcomes. Our findings further support this evidence by identifying preprocedural atorvastatin dosage as an independent predictor of myocardial hypoperfusion, highlighting intensive statin pretreatment as a potentially modifiable strategy. Balloon dilation is a key step in primary PCI for STEMI, compressing thrombus and atherosclerotic plaque to restore coronary blood flow and prepare the vessel for stent implantation. However, rapid balloon deflation may cause abrupt elastic recoil of the vessel wall, dislodging plaque or thrombotic debris and leading to distal embolization (21), which can impair microvascular perfusion and increase the risk of myocardial hypoperfusion after PCI. In contrast, slow balloon deflation, involving gradual pressure reduction, may attenuate vessel recoil, reduce distal embolization, and facilitate recovery of the coronary microcirculation, thereby improving myocardial perfusion (22). Additionally, slow deflation may exert a protective effect similar to ischemic conditioning by mitigating the suction effect associated with rapid pressure release and reducing reperfusion injury. Nevertheless, prolonged balloon deflation may increase afterload and potentially affect left ventricular function, and its long-term clinical impact warrants further investigation (23).

Beyond procedural and therapeutic factors, several biological markers were also identified as independent predictors of myocardial hypoperfusion in the present study. Red cell distribution width (RDW) reflects the heterogeneity of erythrocyte volume in peripheral blood and is commonly considered an indicator of impaired erythrocyte deformability (24, 25). After primary PCI in patients with STEMI, the coronary microvasculature remains relatively narrow, and erythrocytes with reduced deformability may have difficulty passing through the microcirculation, potentially leading to disturbances in myocardial blood flow and contributing to hypoperfusion (26). In addition, elevated RDW has been suggested to be associated with endothelial dysfunction by reducing nitric oxide bioavailability and promoting endothelin-1 release, thereby weakening microvascular vasodilation and potentially compromising myocardial perfusion after PCI (27, 28). However, the precise mechanisms underlying the association between RDW and myocardial hypoperfusion remain incompletely understood. Monoamine oxidase (MAO) is an enzyme involved in the oxidative metabolism of monoamines and is an important source of reactive oxygen species in cardiovascular tissues (29, 30). Elevated MAO activity may increase oxidative stress and has been suggested to contribute to myocardial injury and microvascular dysfunction during ischemia–reperfusion (31). In addition, abnormal MAO activity may influence the expression of vasoactive substances such as angiotensin and endothelin, which could further stimulate nicotinamide adenine dinucleotide phosphate oxidase activity and increase reactive oxygen species production. These processes may accelerate endothelial injury, disrupt coronary microcirculation, and potentially impair myocardial perfusion after PCI (32). Nevertheless, the exact mechanisms linking MAO activity to myocardial hypoperfusion require further investigation.

Beyond identifying clinically relevant predictors, the present study possesses several methodological strengths with important clinical implications. First, the combined use of Boruta and LASSO feature selection enhanced the robustness of variable screening by reducing the risk of overfitting and improving model stability. This stepwise approach allowed for a more reliable identification of independent predictors associated with myocardial hypoperfusion. Second, the prediction model demonstrated favorable performance, with good discrimination, calibration, and clinical utility across both the training and validation cohorts. These findings suggest that the model provides accurate risk estimation and may serve as a practical tool for individualized risk stratification. Importantly, the predictors incorporated into the nomogram are routinely available in clinical practice, enabling convenient application without requiring additional testing or complex calculations. Early identification of high-risk patients may provide a critical window for timely intervention, optimization of peri-procedural management, and ultimately improved myocardial perfusion and clinical outcomes. The present findings may have important clinical implications for the management of patients with STEMI undergoing primary PCI. Early identification of individuals at high risk for myocardial hypoperfusion may allow clinicians to implement targeted preventive strategies, optimize peri-procedural management, and enhance microvascular perfusion. Notably, several predictors identified in this study represent potentially modifiable factors, such as ischemic time, statin pretreatment, and balloon deflation strategy. Timely reperfusion, intensive statin therapy, and appropriate procedural techniques may therefore help mitigate the risk of hypoperfusion and improve myocardial recovery. Furthermore, the nomogram developed in this study integrates routinely accessible clinical and laboratory parameters, supporting its feasibility for real-world application. By facilitating individualized risk assessment, this tool may contribute to more precise clinical decision-making and ultimately improve patient outcomes. In addition, in some high-risk patients, especially those with cardiogenic shock, impaired perfusion may partly reflect systemic hypoperfusion caused by reduced cardiac output rather than a purely coronary or microvascular phenomenon. Recent studies have also suggested that antithrombotic strategies, including the choice of P2Y12 inhibitor and the use of post-procedure anticoagulation, may influence prognosis in STEMI patients undergoing primary PCI (33, 34). Therefore, future studies may further evaluate whether incorporating these treatment-related factors could improve prediction of myocardial hypoperfusion and related outcomes. Several limitations of this study should be acknowledged. First, although multiple clinically relevant variables were included, the potential influence of residual confounding from unmeasured factors cannot be entirely excluded. Second, the present study primarily focused on short-term myocardial perfusion after PCI, while long-term clinical outcomes were not assessed. Future studies with extended follow-up are needed to determine the prognostic value of the model for long-term cardiovascular events. Third, myocardial perfusion in this study was assessed using the TIMI flow grade based on coronary angiography. Although this method is widely used in clinical practice, it may not fully reflect myocardial microvascular perfusion. Advanced imaging modalities such as cardiac magnetic resonance or myocardial radionuclide imaging could provide more accurate evaluation of myocardial perfusion and microvascular obstruction. Future studies incorporating these imaging techniques may further improve the assessment of myocardial perfusion after PCI. Fourth, some variables in the multivariable logistic regression analysis showed relatively wide confidence intervals, which may reflect limited precision of the effect estimates. Therefore, these associations should be interpreted with caution. Finally, this study was retrospective in nature and conducted at a single center, which may introduce selection bias and limit the generalizability of the findings to other populations or clinical settings. In addition, external validation was not performed in an independent cohort. Therefore, prospective multicenter studies with larger sample sizes and independent external validation cohorts are warranted to further confirm the robustness, stability, and generalizability of the proposed prediction model.

## 5 Conclusion

In conclusion, this study identified key determinants of myocardial hypoperfusion following primary PCI in patients with STEMI and developed a nomogram to enable individualized risk prediction. Time from onset to primary PCI, atorvastatin dose before PCI, balloon deflation method during PCI, RDW, and MAO levels were found to be independent predictors. The model demonstrated favorable discrimination, calibration, and clinical utility, suggesting reliable predictive performance. By integrating routinely available clinical and laboratory parameters, this tool may facilitate early risk stratification, support targeted preventive strategies, and contribute to improved myocardial perfusion and clinical outcomes. Further multicenter prospective studies are warranted to validate these findings.

## Data Availability

The raw data supporting the conclusions of this article will be made available by the authors, without undue reservation.
